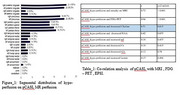# Harnessing Pcasl : Unveiling Cerebral Hemodynamics And Advancing Biomarkers In Alzheimer's Disease ‐ A Simultaneous MR/PET Study

**DOI:** 10.1002/alz70856_098738

**Published:** 2025-12-24

**Authors:** Sandhya Mangalore

**Affiliations:** ^1^ National Institute of Mental Health and Neurosciences, Bengaluru, India

## Abstract

**Background:**

pCASL is a non‐invasive method to measure CBF, which is essential in understandingthe pathophysiology of AD.

**Method:**

A prospective cross‐sectional observational study was conducted from July 2021 to July 2023, including 25 patients clinically diagnosed with AD. We established pCASL MRI study protocol as part oftheir MRI examination, and correlating these findings with other imaging modalities. Cerebral parcellationwas done based on Cortex_ID.

**Result:**

Our results showed that similar to brain parenchymal atrophy, hypo‐perfusion on pCASL MRperfusion was predominantly observed in temporal and parietal lobes. Hypo‐perfusion was mainlyobserved in, the right posterior cingulate gyrus in all patients along with a significant involvement of leftposterior cingulate gyrus, precuneus, superior and inferior parietal lobes aligning with known patterns ofAD‐related neurodegeneration.

**Conclusion:**

The study signifies the potential of pCASL in the diagnosis of AD. It confirms the prevalentinvolvement of parietal and temporal lobes, demonstrating hypo‐perfusion and hypo‐metabolism, which isconsistent with previous studies indicating DMN disruption. Approximately 60‐75% of patients exhibitedhypo‐perfusion in the parietal lobes, supporting the role of pCASL as a reliable indicator of AD‐relatedimpairments in visuospatial function and attention. Our research emphasizes the need for standardizedpCASL protocols to enable consistent and comparable results across studies. The integration of pCASL withother imaging modalities and biomarkers could provide a comprehensive understanding of ADpathophysiology. This multimodal approach, coupled with larger and more diverse study populations, maypave the way for the identification of robust CBF‐based biomarkers, enhancing early diagnosis.